# Brief Report: Anomalous Neural Deactivations and Functional Connectivity During Receptive Language in Autism Spectrum Disorder: A Functional MRI Study

**DOI:** 10.1007/s10803-014-2344-y

**Published:** 2014-12-20

**Authors:** Ariel Karten, Joy Hirsch

**Affiliations:** 1 Brain Function Laboratory, Departments of Psychiatry and Neurobiology, Yale University School of Medicine, 300 George Street, Suite 902, New Haven, CT 06511 USA; 2 Departments of Psychiatry and Neurobiology, Yale University School of Medicine, 300 George Street, Suite 902, New Haven, CT 06511 USA; 3 Stony Brook University School of Medicine, 101 Nicolls Rd., Stony Brook, NY 11794 USA

**Keywords:** Functional magnetic resonance imaging (fMRI), Functional connectivity, Psychophysiological interactions (PPI), Negative BOLD response (NBR), Neural inhibition, Autism, Receptive language processing

## Abstract

Neural mechanisms that underlie language disability in autism spectrum disorder (ASD) have been associated with reduced excitatory processes observed as positive blood oxygen level dependent (BOLD) responses. However, negative BOLD responses (NBR) associated with language and inhibitory processes have been less studied in ASD. In this study, functional magnetic resonance imaging showed that the NBR in ASD participants was reduced during passive listening to spoken narratives compared to control participants. Further, functional connectivity between the superior temporal gyrus and regions that exhibited a NBR during receptive language in control participants was increased in ASD participants. These findings extend models for receptive language disability in ASD to include anomalous neural deactivations and connectivity consistent with reduced or poorly modulated inhibitory processes.

## Introduction

Autism spectrum disorder (ASD) is a prevalent neurodevelopmental disorder characterized by a spectrum of language and communication deficits without known mechanisms. It has been suggested that ASD may involve anomalous inhibitory neural processes in the brain (Gogolla et al. 2009; Hussman 2001; Rubenstein and Merzenich 2003; Uhlhaas and Singer 2012; Yizhar et al. 2011), however, evidence for these models remains an active area of investigation.

Functional imaging studies of auditory receptive language based on the positive blood oxygen level dependent (BOLD) response (PBR) have shown that the canonical language areas, such as Wernicke’s and Broca’s areas, are less activated in participants with ASD relative to typical controls (Gervais et al. 2004; Lai et al. 2011, 2012). However, the negative BOLD response (NBR) has not been examined in this context. In general, the PBR is interpreted as an engagement or excitation of a neural substrate (Logothetis et al. 2001), whereas the NBR is thought to reflect alternative signal processes that have been associated with inhibitory or suppressive mechanisms (Smith et al. 2004; Amedi et al. 2005; Shmuel et al. 2002, 2006; Wade 2002). Consistent with this interpretation, the concentration of the inhibitory neurotransmitter gamma-aminobutyric acid (GABA) has been shown to be inversely correlated to the strength of the PBR (Chen et al. 2005; Muthukumaraswamy et al. 2009), while directly correlated to an increase in the NBR (Northoff et al. 2007).

Although much emphasis has been placed on the PBR and its ability to reveal neural activity and inter-area connections during a given task, the NBR and its relationship to inhibition has recently emerged as an active topic of investigation. In particular, a specific constellation of regions known as the default mode network (DMN) has been observed to deactivate during cognitive tasks (Gusnard et al. 2001; Raichle et al. 2001), visual perception (Karten et al. 2013), and language processing (Seghier and Price 2012). Regions previously identified as involved in attention and working memory, have also been associated with the NBR during language studies (Diaz and McCarthy 2009; Seghier and Price 2009). However, language disability in ASD has not been previously related to the NBR and putative inhibitory processes as predicted by the above. Despite previous implications of atypical GABA and neural inhibitory processes in ASD (Gogolla et al. 2009; Hussman 2001; Rubenstein and Merzenich 2003; Uhlhaas and Singer 2012; Yizhar et al. 2011), it is not well understood how such anomalies would impact large-scale neural networks engaged during language functions. As such, the NBR presents a unique opportunity to investigate both regional responses and global neural networks involved in putative inhibitory processes associated with receptive language functions in ASD. In this study, we test the hypothesis that receptive language-related function in ASD is associated with reduced NBRs and altered functional connectivity consistent with anomalous inhibitory processes.

## Materials and Methods

Imaging data for this study have been reported previously as a proposed diagnostic for ASD using speech-induced activation in Wernicke’s area as a basis for a biomarker to detect ASD (Lai et al. 2011), and also as a comparison of speech and song-related mechanisms in ASD showing that song was more effective than spoken narratives to activate language sensitive systems (Lai et al. 2012). The raw data and the analysis of the PBR have been employed in the previous studies of Lai et al. (2011, 2012), however the analysis of the NBR and associated functional connectivity are novel analyses and have not been investigated or reported previously. Informed written consent, based on the guidelines established by the Columbia University Medical Center Institutional Review Board, was acquired from both parents of each child. Twelve ASD participants (mean age = 12.40 years, SD = 4.70, range = 7.01–22.47 years; males = 10; right-handed = 10), and twelve healthy controls (mean age = 12.48 years, SD = 3.80, range = 7.85–17.78 years; males = 8; right-handed = 10) participated in the study (Table 1, 2). These samples were matched with respect to age (ASD mean age 12.4 ± 4.7 vs. control mean age 12.48 ± 3.8) and handedness (ten right handed). A Chi square test of gender distribution failed to show a difference in the gender composition of the groups (*p* < 0.15). Medical examinations confirmed that participants with ASD were not visually or auditorily impaired. Participants with ASD were diagnosed based on the Diagnostic and Statistical Manual of Mental Disorders-IV (DSM-IV; American Psychiatric Association 1994) and the Autism Diagnostic Interview-Revised (ADI-R; Lord et al. 1994). A diagnosis of ASD based on the ADI-R is given to patients who score higher than a ten on the social subscale, an eight on the language and communication subscale, and a three on the repetitive behavior subscale. On average, the participants scored 20.17 (SD = 2.08) on the social subscale, 18.50 (SD = 2.84) on the language and communication subscale, and 5.92 (SD = 1.16) on the repetitive behavior subscale, all of which are well above the minimum requirements for a diagnosis of ASD (Table 1). Physician observations during a 30 min free play session determined that the participants with ASD spontaneously verbalized an average of 16.29 words (SD = 42.70, median = 4) and in response to a question spoke on average 46.4 words (SD = 76.16, median = 14) further confirming the severity of their communication disabilities. Control participants were without a diagnosis of ASD, neurological disorders, or siblings with ASD. Although a medical examination was not required for eligibility for the study, parents affirmed that their child was not on a current medication for a hyperactive condition or any other psychiatric or neurological condition. Behavioral information based on scholastic achievement and age appropriate grade level was also used to confirm that control participants were developmentally age-typical.

**Table 1:** ASD participants

Participant no./sex	Age at imaging	Handedness	ADI-R social	ADI-R language	ADI-R repetitive behavior
1 M	16.72	Right	20	17	6
2 M	7.01	Ambi	22	18	6
3 M	10.85	Right	22	17	6
4 M	22.47	Right	21	22	8
5 F	8.38	Right	21	20	5
6 M	9.10	Left	21	18	8
7 M	16.56	Right	19	22	6
8 M	9.21	Right	19	20	5
9 M	13.39	Right	19	19	6
10 F	15.65	Right	17	16	5
11 M	7.41	Right	17	12	4
12 M	12.09	Right	24	21	6
Mean	12.40 ± 4.70		20.17 ± 2.08	18.50 ± 2.84	5.92 ± 1.16

**Table 2:** Control participants

Participant no./sex	Age at imaging	Handedness
1 M	16.84	Left
2 F	7.90	Right
3 M	11.1	Right
4 M	17.78	Right
5 M	8.95	Left
6 M	9.64	Right
7 F	17.51	Right
8 M	9.64	Right
9 M	13.55	Right
10 F	15.93	Right
11 F	7.85	Right
12 M	13.07	Right
Mean	12.48 ± 3.80	

The experimental paradigm was composed of two imaging runs each totaling 2 min and 29 s consisting of an initial 24 s period of background scanner noise followed by four 15 s epochs of passive listening to recorded speech by each participant’s parents interspersed with 15 s rest epochs. The listening task was “passive” in that the participants were asked to listen to the incoming auditory stimulus without requirement to respond. Auditory stimulation was delivered to the participants via MRI safe headphones. A muted preselected video was played throughout the duration of the run, including during the passive listening stimulation, either on a rear-projection screen or on MRI compatible goggles, in order to encourage minimal head movements. Comparisons of fMRI activity between the ASD participants in this study and sedated ASD participants who were exposed to the same auditory conditions (but without the muted video) showed no differences with or without the video consistent with there being no measureable effects due to viewing the video (Lai et al. 2011, 2012). The auditory narrative was pre-recorded by a parent who was instructed to address the participant directly in a personal and familiar manner. Additionally, all parents were asked to talk about the same topics and compliance was confirmed in all cases by the research team. These instructions were intended to assure that the parental recordings were equally familiar and salient to all participants, and independent reviewers confirmed that the recordings for the ASD and control participants could not be distinguished. That is, reviewers could not sort the narratives into the two groups, which is consistent with the presentation of similar recordings for each. The recordings were composed of the same topics (i.e. being in the scanner, recent events, and family plans after the imaging session). This design was selected in order to increase task compliance in young children and ASD participants, whereby the parent’s voice would be a meaningful and calming influence during the scanning session. Additionally, the stimuli were power-normalized thus ensuring similar acoustic properties across all participants.

Passive auditory stimulation using spoken narratives has been shown to activate neural substrates of the language system (Hirsch et al. 2000), and is used with children in clinical settings to map the locations of language-sensitive regions in preparation for neurosurgical procedures (Souweidane et al. 1999). Due to this prior validation, a similar passive listening paradigm was chosen for this study as the severity of the language impairment in the ASD participants ruled out options for a more complex interactive task and options for performance evaluations.

Functional imaging of the control and ASD participants was carried out on a research-dedicated 1.5T GE Medical Systems (Milwaukee, WI, USA) Twin Speed MRI scanner located in the Columbia University fMRI Research Center, New York, NY. Whole brain functional images were acquired using an ecoplanar T2*-weighted gradient echo sequence (TR = 3,000 ms, echo time = 51 ms, flip angle = 83°) with 27 contiguous axial slices acquired along the anterior–posterior commissure plane (FoV = 192 × 192 mm, array size = 128 × 128, spatial resolution = 1.56 × 1.56 × 4.5 mm).

Image pre-processing and statistical analysis was performed using SPM8 software (Wellcome Department of Cognitive Neurology, University College London, UK). Images were slice-timing corrected and spatially realigned to the first volume of the first run. The scans were co-registered with the mean realigned EPI image. Normalization parameters were applied to a standard template image, and combined realignment and inverse co-registration normalization parameters were applied to the functional images. Images were smoothed with a Gaussian kernel of 8.0 × 8.0 × 8.0 mm full-width at half-maximum, and a 128 s temporal high-pass filter was applied.

Task onset times were convolved with the canonical hemodynamic response function (HRF). Contrasts of resulting beta estimates (“Task” > “Baseline”) were passed to second level random effects (RFX) analyses (one-sample *t* tests). Beta estimates from each run were also passed to a second level RFX analysis (two-sample *t* test) in order to determine activations and deactivations common to each of the groups. The General Linear Model, yields either positive or negative beta values depending upon the polarity of the raw data. These signals are differentiated by their polarity as either a positive canonical HRF or a negative canonical HRF, respectively. Locations of regions of interest, ROIs, selected a priori were defined based on the group activations and were used to create seeds for the psychophysiological interaction (PPI) analysis of functional connectivity. To control for multiple comparisons, cluster-extent thresholding was applied using an uncorrected cutoff *p* ≤ 0.005 and cluster size threshold of 150 contiguous voxels resulting in an effective corrected threshold of *p* ≤ 0.05. This cluster threshold was determined by 10,000 Monte Carlo simulations of whole-brain fMRI data with the respective parameters of this study using AlphaSim in AFNI (v2009).

Functional connectivity, based on the PPI (Friston et al. 1997; Friston 2011), to measure the extent to which brain regions were differentially correlated between conditions, was employed to compare ASD and control participants during the receptive language task. A bilateral cluster of the superior temporal gyrus, STG, activity (centered at x = ± 54 y = −22 z = 6) was used to create a seed based on the common activity of both the ASD and control groups using the Marsbar Toolbox (http://marsbar.sourceforge.net/). The STG was first defined anatomically using the Wake Forest University PickAtlas (Maldjian et al. 2003; Lancaster et al. 1997) based on individual structural images, and then further refined in group analysis based on the common of activity of both the ASD and control groups in group analysis. The STG was chosen a priori as the seed region because of known engagement during receptive language (Binder et al. 1994) and was the only common auditory processing region in both the control and ASD groups. BOLD signals throughout the whole-brain were regressed on a voxel-wise basis against the product of the time course of the seed and the vector of the psychological variable of interest (epochs of auditory stimulation vs. baseline). Resulting beta maps, within each run and averaged across both runs, were subsequently passed to second level random effects analysis (one sample *t* test). General linear models that were used to extract seed region activity and to estimate PPI results included additional nuisance regressors, i.e. six motion parameters, mean white-matter, and mean csf signal.

## Results

### Positive BOLD Response (PBR)

**Fig. 1:**
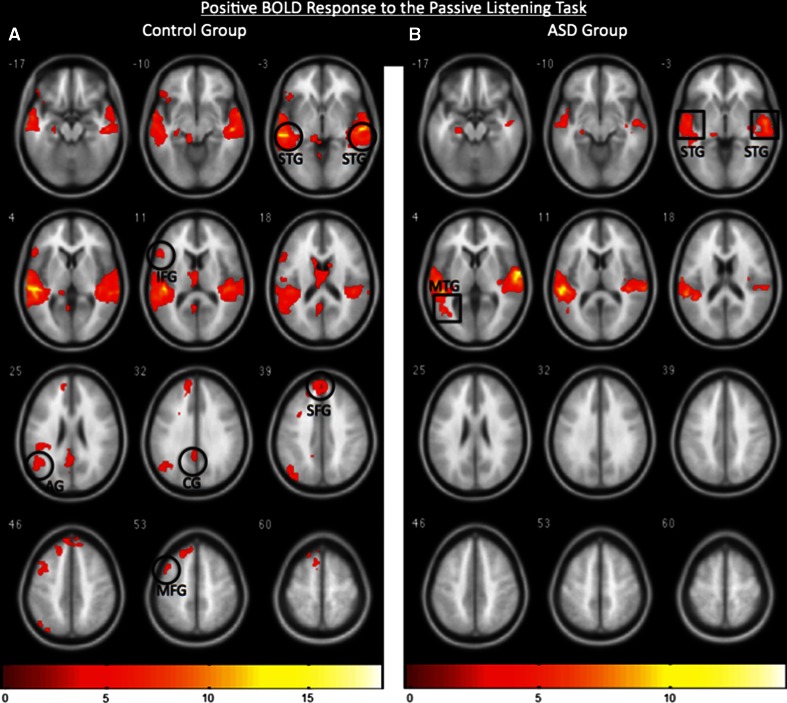
**a** Positive BOLD-related fMRI activity associated with the control group (n = 12) showing canonical language-sensitive regions including the superior temporal gyrus (STG), inferior frontal gyrus (IFG), superior frontal gyrus (SFG), middle frontal gyrus (MFG), angular gyrus (AG), and cingulated gyrus (CG). **b** Positive BOLD-related fMRI activity associated with the ASD group (n = 12) showing activation of the STG and MTG. Images are thresholded at *p* < 0.005, k = 150 for an effective cluster correction of *p* < 0.05, and *color bars* indicate z-scores. The *right* and *left* sides of the figure correspond to the *right* and *left* hemispheres, respectively

As expected for typical control participants during the passive language task, the pattern of the PBR included the superior temporal gyrus (STG), left inferior frontal gyrus (IFG), middle frontal gyrus (MFG), superior frontal gyrus (SFG), left angular gyrus (AG), and cingulate gyrus (CG) (Fig. 1a; *p* < 0.005, k = 150 for an effective cluster correction of *p* < 0.05). ASD participants also showed activation in the STG and middle temporal gyrus (MTG), however, there was no evidence for activations in the frontal and parietal language areas (Fig. 1b; *p* < 0.005, k = 150 for an effective cluster correction of *p* < 0.05). These findings are consistent with previous reports of reduced neural activations, represented by the PBR, in response to a language task for ASD participants (Gervais et al. 2004; Lai et al. 2012), and are included here for comparison with the NBR.

### Negative BOLD Response (NBR)

**Fig. 2:**
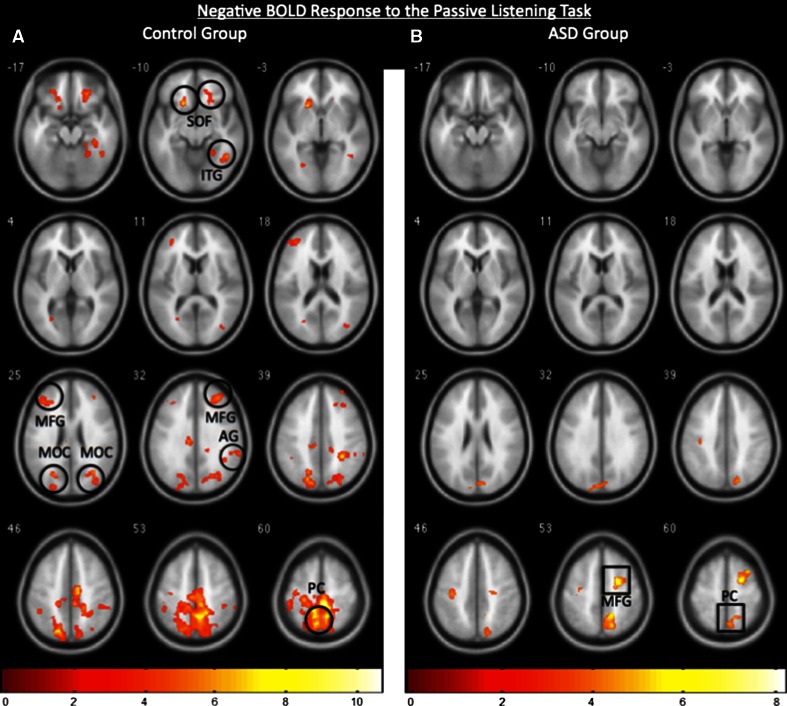
**a** Negative BOLD-related fMRI activity for the control group (n = 12) showing deactivations in the superior orbitofrontal cortex (SOF), inferior temporal gyrus (ITG), middle frontal gyrus (MFG), middle occipital cortex (MOC), angular gyrus (AG), and precuneus (PC). **b** Negative BOLD-related fMRI activity for the ASD group (n = 12) showing deactivations of the PC and MFG. Note the relative difference in extent and magnitude between the two groups in their respective negative BOLD responses. Images are thresholded at *p* < 0.005, k = 150 for an effective cluster correction of *p* < 0.05, and *color bars* indicate z-scores. The *right* and *left* sides of the figure correspond to the *right* and *left* hemispheres, respectively

**Fig. 3:**
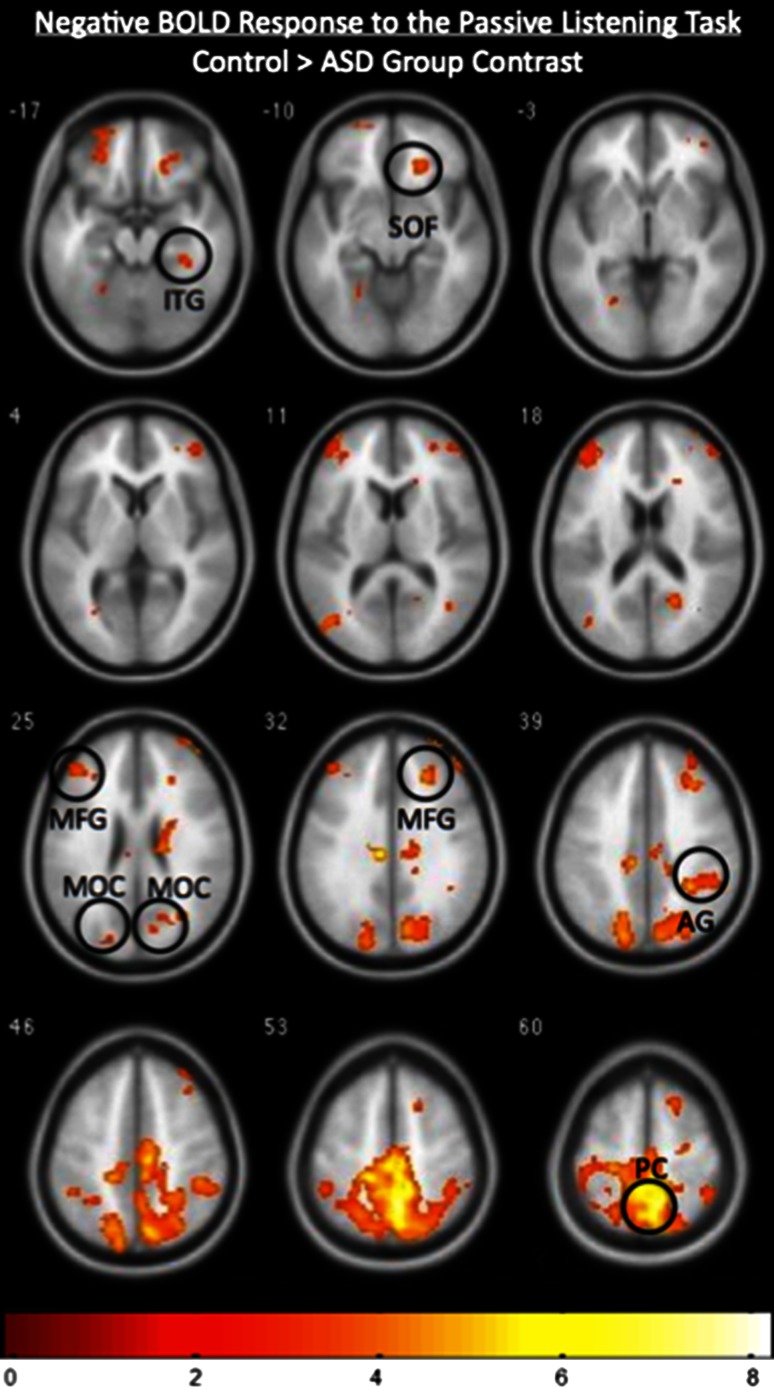
Group contrast of the negative BOLD-related fMRI activity, showing regions that exhibited a greater NBR in the control group (N = 12) than the ASD group (N = 12). The superior orbitofrontal cortex (SOF), inferior temporal gyrus (ITG), middle frontal gyrus (MFG), middle occipital cortex (MOC), angular gyrus (AG), and precuneus (PC) all display a greater NBR in the control group than in the ASD group. The image is thresholded at *p* < 0.005, k = 150 for an effective cluster correction of *p* < 0.05, and the *color bar* indicates z-scores. The *right* and *left* sides of the figure correspond to the *right* and *left* hemispheres, respectively

In the case of control participants, the pattern of the NBR during the passive listening task revealed robust deactivations of the precuneus (PC), superior orbitofrontal cortex (SOF), inferior temporal gyrus (ITG), middle occipital cortex (MOC), right AG and MFG during the auditory stimulation at *p* < 0.005, k = 150 for an effective cluster correction of *p* < 0.05 (Fig. 2a). However, the NBR for the ASD participants was limited to deactivations of the PC and MFG at *p* < 0.005, k = 150 for an effective cluster correction of *p* < 0.05 (Fig. 2b). Group contrast of the NBR for the control group > ASD group confirmed that the NBR observed in the PC, SOF, ITG, MOC, AG, and MFG was significantly greater in controls than in the ASD participants at *p* < 0.005 and k = 150 (Fig. 3).

### Event-Triggered Average Signals

**Fig. 4:**
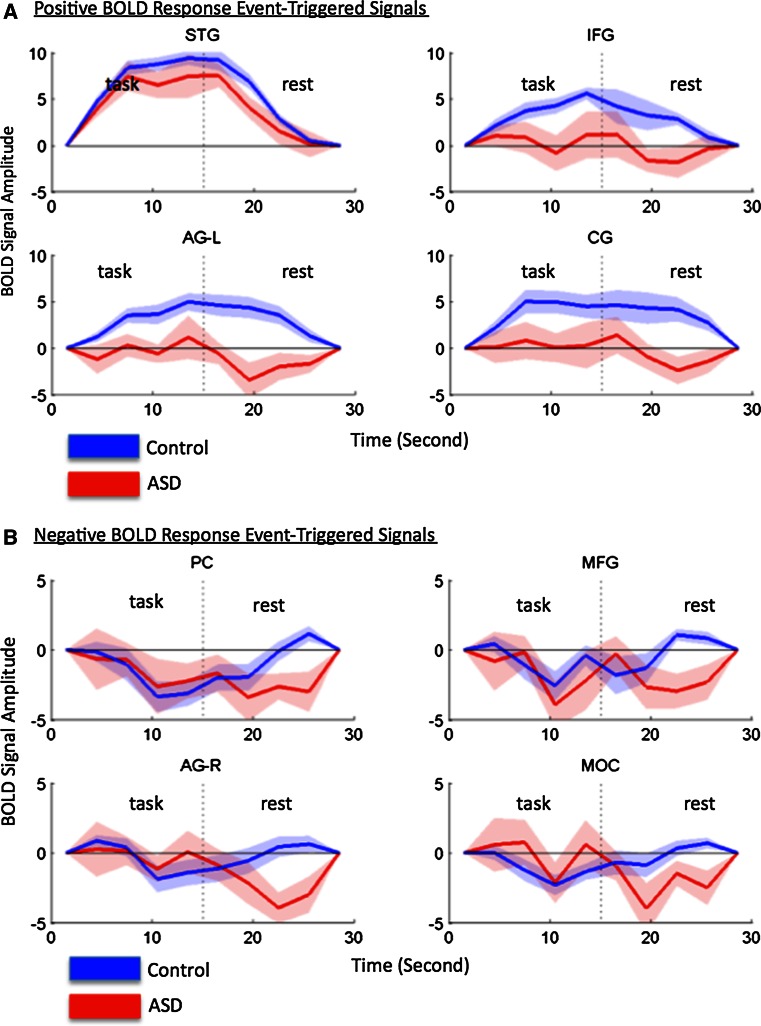
**a** Event-triggered averaged signals from the superior temporal gyrus (STG), inferior frontal gyrus (IFG), left angular gyrus (AG-L), and cingulate gyrus (CG), all of which displayed a PBR relative to the baseline in response to being presented with speech stimulation. The *blue* *line* corresponds to the signal of the control participants, and the *red line* corresponds to the signal of the ASD participants. The *blue* and *red* *shadows around the lines* reflect ± 1 SE of the mean. **b** Event-triggered averaged signals from the precuneus (PC), middle frontal gyrus (MFG), right angular gyrus (AG-R), and middle occipital gyrus (MOC), all of which displayed a NBR relative to the baseline in response to being presented with speech stimulation. The *blue* *line* corresponds to the signal of the control participants, and the *red* *line* corresponds to the signal of the ASD participants. The *blue* and *red* *shadows around the lines* reflect ± 1 SE of the mean

Further insight into the PBR and NBR for both groups is provided by the event-triggered averaged signals (Fig. 4a, b). The BOLD signals for all task and rest epochs were averaged for control and ASD groups for representative regions of interest for the PBR (Fig. 4a) and for the NBR (Fig. 4b). Consistent with the “heat” map representations (Fig. 1a, b), the PBR in the STG for participants with ASD (red) is present but depressed relative to the controls (blue), and the signals in the left IFG, left AG, and CG were not significantly different from baseline for the ASD participants. In the case of the NBR (Fig. 4b), representative regions including the PC, MFG, right AG, and MOC were well fit by the predicted negative canonical HRF for the control participants (Table 3). However, in the case of the ASD participants the HRF based on the NBR event-triggered data was no different than that of a baseline signal (Table 3). The *p* values in Table 3 reflect the goodness-of-fit between the observed event-triggered signal and the modeled HRF for each ROI. As indicated by the *p* values and illustrated by Fig. 4a and b, the canonical HRF is well fit by all control ROIs with both positive and negative signals. However in the ASD group the canonical HRF is only well fit by the PBR in the STG.

**Table 3:** *p* values indicating the goodness-of-fit between the event-triggered response and the canonical HRF for representative PBR and NBR regions

	Control (n = 12)	ASD (n = 12)
PBR region		
STG	0.0001	0.0008
IFG	0.0001	0.8274
AG (left)	0.0001	0.8770
CG	0.0022	0.3050
NBR region		
PC	0.0002	0.1410
MFG	0.0109	0.2504
AG (right)	0.0060	0.9421
MOC	0.0046	0.5665

### Functional Connectivity

**Fig. 5:**
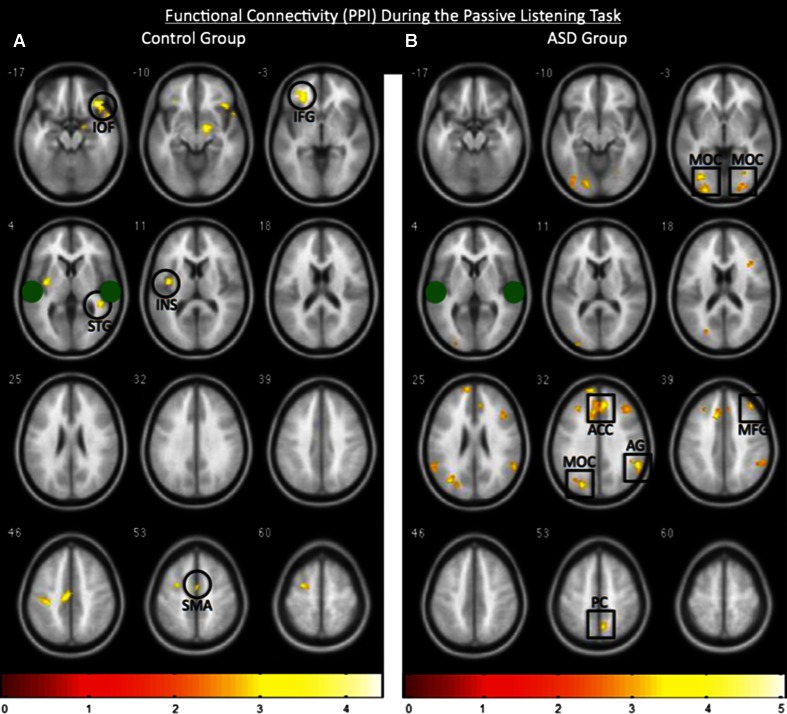
Functional connectivity (PPI) seeded with the bilateral STG clusters. The approximate centers of the seeds are indicated by the green filled circles. **a** The control group (n = 12) displayed a heightened correlation between the seed and inferior orbitofrontal cortex (IOF), inferior frontal gyrus (IFG), insula (INS), superior temporal gyrus (STG), and supplementary motor area (SMA) whereas **b** the ASD participants (n = 12) exhibited a heightened correlation between the seed and middle occipital cortex (MOC), middle frontal gyrus (MFG), anterior cingulate cortex (ACC), angular gyrus (AG), and, precuneus (PC) most of which normally deactivate in healthy controls. Figures are group results displayed at a statistical threshold of *p* < 0.01, and *color bars* indicate z-scores. The *right* and *left* sides of the figure correspond to the *right* and *left* hemispheres, respectively

In healthy controls, relative to the ASD group, functional connectivity seeded with the STG, revealed increased connectivity with the language-sensitive areas including, the IFG, STG, insula (INS), inferior orbitofrontal cortex (IOF), and supplementary motor area (SMA) as expected during the task (Fig. 5a; *p* < 0.01). However, relative to the controls the ASD participants showed increased connectivity between the STG and the MOC, AG, MFG, PC, and anterior cingulate cortex (ACC) during the task (Fig. 5b; *p* < 0.01) all of which, other than the ACC, are regions that deactivated (negative BOLD response) during the receptive language task in the control participants, and, notably, include components of the DMN (Gusnard et al. 2001; Karten et al. 2013; Raichle et al. 2001).

## Discussion

Here we show that ASD participants demonstrate an atypical NBR relative to that of healthy controls during passive listening to spoken narratives. These signal differences between the control and ASD participants extend the known differences for speech processing in the ASD brain beyond activation to also include deactivation, and are consistent with the hypothesis that language disability in ASD is also related to a deficiency of inhibitory processes as indicated by the NBR. Further, in healthy developmentally typical controls, the STG is functionally connected to other known language-sensitive regions. However in ASD participants the connectivity appears to be altered, and is increased to many of the regions that normally deactivate during the task in healthy controls. Together these findings suggest that models for language disability in the ASD brain include atypical responses of oppositional excitatory/inhibitory processes and functional connectivity.

While the functional role of neural inhibition is still poorly understood, distributed patterns of neural activations and deactivations have been implicated in general attention and cognitive processing (Gusnard et al. 2001; Raichle et al. 2001), and comprehension of spoken narratives (Rodriguez-Moreno et al. 2014). An anti-correlation has been observed between the default mode and frontoparietal networks, wherein as one network is activated the other deactivates (Fox et al. 2005; Uddin et al. 2009). Thus, an intrinsic oppositional organization includes neural deactivations, and numerous neurological disorders such as schizophrenia (Pomarol-Clotet et al. 2008), attention deficit hyperactivity disorder (Fassbender et al. 2009), and ASD (Kennedy et al. 2006) have been associated with these default mode processes. Findings in this paper extend a role for neural deactivations in function-specific deficits including receptive language in ASD.

It has been proposed that ASD may be related to low levels of GABA in the brain (Hussman 2001). This hypothesis has been supported by an atypical excitatory/inhibitory ratio observed in ASD neural systems (Gogolla et al. 2009; Rubenstein and Merzenich 2003; Yizhar et al. 2011). Further supporting the GABA hypothesis, are animal models that exhibit ASD-like social and developmental impairments when the gabrb3 gene, which codes for the GABA_A_ receptor, is knocked out (DeLorey et al. 2008). Consistent with the animal models, evidence of a downregulation of the GABA_A_ receptor has been shown in human ASD participants (Fatemi et al. 2009). Additionally it has been proposed that many of the symptoms seen in ASD may be related to an overabundance of incoming sensory information (Pritchard et al. 1987; Rogers and Ozonoff 2005), which given the current findings, is consistent with a deficiency of neural suppression to regulate sensory input.

The NBR has been directly correlated to GABA levels in the brain (Northoff et al. 2007), suggesting a possible link between anomalous language-related functions, the NBR, and levels of GABA. The finding that the event-triggered averaged NBRs were more variable than comparable control NBRs, and therefore the HRF was no different than a baseline signal, (Table 3), is consistent with atypical inhibitory processes. The additional and unanticipated finding in the ASD participants, that during passive listening the STG is functionally connected to many regions that would normally deactivate in healthy controls during the same task, supports the notion of a systems-level abnormality. Together these findings are consistent with widespread atypical inhibitory processes in the ASD brain, and motivate further related research.

These fMRI findings including the NBR and functional connectivity extend models of neural inhibition and ASD to a global network level. Our findings contribute additional specification regarding the neural substrates in ASD presumed to function in an atypical manner during receptive language. The localization of deficient inhibitory processes during passive listening to spoken narratives may also have significant clinical implications for understanding the mechanisms underlying the disorder and the eventual development of targeted therapies.

This study is limited to language impaired ASD participants who were not matched in IQ with the control participants. Therefore, it cannot be ruled out that variations in IQ may contribute to the results. Additionally, in order to rule out that gender or handedness influenced the results, analyses were performed on only the right-handed participants and on only the male participants. The additional analyses confirmed that the results from these sub-groups did not differ from the complete data set. Results from these subsets are consistent with the conclusion that gender and handedness were not confounds in the study. The fMRI task was passive listening due to the limited ability of the ASD participants to perform a volitional response, and therefore no correlations can be made between performance on a task and degree of impairment. Future studies may aim to use a less impaired ASD group capable of providing a performance measure and a more interactive task, thus allowing for the investigation of variations in neural inhibition as measured by the NBR and the degree of receptive language impairment.
